# Unexpectedly High Prevalence of Low Alpha-Galactosidase A Enzyme Activity in Patients with Focal Segmental Glomerulosclerosis

**DOI:** 10.6061/clinics/2020/e1811

**Published:** 2020-09-23

**Authors:** Nuri Baris Hasbal, Feyza Bayrakdar Caglayan, Tamer Sakaci, Elbis Ahbap, Yener Koc, Mustafa Sevinc, Zuhal Atan Ucar, Abdulkadir Unsal, Taner Basturk

**Affiliations:** I Nephrology Unit, Hakkari State Hospital, Hakkari, Turkey; II Department of Nephrology Istanbul Taksim Training and Research Hospital, Istanbul, Turkey; III Department of Nephrology, Sisli Hamidiye Etfal Training and Research Hospital, Istanbul, Turkey; IV Division of Nephrology, Department of Internal Medicine, Medical Faculty, Cumhuriyet University, Sivas, Turkey; V Division of Nephrology, Department of Internal Medicine, Medical Faculty, Demiroglu Bilim University, Istanbul, Turkey; VI Department of Internal Medicine, Faculty of Medicine, Health Sciences University, Istanbul, Turkey

**Keywords:** Alpha-Galactosidase A, Focal Segmental Glomerulosclerosis, LysoGb3

## Abstract

**OBJECTIVES:**

Fabry disease (FD) is a rare disease associated with sphingolipid accumulation. Sphingolipids are components of plasma membranes that are important in podocyte function and accumulate in various glomerular diseases such as focal segmental glomerulosclerosis (FSGS). Both FD and FSGS can cause podocyte damage and are classified as podocytopathies. In this respect, FD and FSGS share the same pathophysiologic pathways. Previous screening studies have shown that a significant proportion of end-stage renal disease (ESRD) patients receiving hemodialysis (HD) have unsuspected FD, and the prevalence of low alpha-galactosidase A (αGLA) enzyme activity in these patients is higher than that in the normal population. We aimed to compare αGLA enzyme activity in patients with biopsy-proven FSGS and ESRD receiving HD.

**METHODS:**

The records of 232 patients [62 FSGS (F/M: 33/29); 170 HD (M/F: 93/79)] were evaluated retrospectively. The screening was performed based on the αGLA enzyme activity on a dried blood spot, with the confirmation of plasma LysoGb3 levels, and the known GLA mutations were tested in patients with low enzyme activities. The two groups were compared using these parameters.

**RESULTS:**

The mean level of αGLA enzyme activity was found to be lower in FSGS patients than in the HD group (2.88±1.2 μmol/L/h *versus* 3.79±1.9 μmol/L/h, *p*<0.001). There was no significant relationship between the two groups with regard to the plasma LysoGb3 levels (2.2±1.22 ng/ml *versus* 1.7±0.66 ng/ml, p: 0.4). In the analysis of GLA mutations, a D313Y mutation [C(937G>T) in exon p] was found in one patient from the FSGS group.

**CONCLUSIONS:**

We found that αGAL activity in patients with FSGS is lower than that in patients undergoing HD. The low enzyme activity in patients with FSGS may be explained by considering the similar pathogenesis of FSGS and FD, which may also lead to sphingolipid deposition and podocyte injury.

## INTRODUCTION

Fabry disease (FD) is characterized by an X-linked (Xq22.1) defect of glycosphingolipid catabolism. *GLA*-coding region mutations that cause deficient α-galactosidase A (αGLA) enzyme activity may result in this disease (1). This deficiency leads to globotriaosylceramide (Gb3 or GL3) deposition in the lysosomes of various cells (2). Renal involvement of the disease is observed in approximately 55% of patients with FD. Glomerular damage, glomerulosclerosis, proteinuria, microalbuminuria, hematuria, renal cysts, and progressive reduction in glomerular filtration rate are common in classical FD (3,4); however, these features may be absent in variant forms of the disease. Patients typically present with late-onset proteinuria and end-stage renal disease (ESRD) in the later years of life (5).

Podocyte and/or two sites of the glomerular basement membrane may be affected in FD. Vascular changes, interstitial fibrosis, tubular atrophy, and focal or diffuse glomerulosclerosis are common chronic histological findings in renal biopsy specimens of subjects with glomerulopathies such as FD. Some patients with FD who have chronic histopathological findings are misdiagnosed as having primary FSGS and exposed to possibly harmful immunosuppressive therapies (6). Additionally, a subgroup of FSGS patients with partial or no response to therapy may be diagnosed wrongly, and in reality, suffer from FD. In this study, we investigated αGLA enzyme activity in patients with biopsy-proven FSGS and undergoing hemodialysis (HD).

## METHODS

The study was presented as a poster presentation at the fifty-fifth Congress of ERA-EDTA, Copenhagen, Denmark.

The records of 62 patients with biopsy-proven FSGS and 170 patients with ESRD undergoing hemodialysis (HD) at the Sisli Hamidiye Etfal Training and Research Hospital, Department of Nephrology, between January 2010 and March 2015, were evaluated retrospectively. For these patients, α-galactosidase A (αGLA) enzyme activity in plasma was measured using the dried blood spot (DBS) method as a source of DNA, *via* tandem mass spectrometry (7). Blood was transferred onto a filter paper, kept at room temperature to dry, and then maintained at 22-24°C until analysis. The enzyme activities were calculated in μmol/L/h. Although there are different cut-off values for low αGLA enzyme activity in the literature (varies from 0.6 to 2.5 µmol/l/h), it was defined as lesser than 1.2 µmol/l/h by Receiver Operating Characteristic testing, which was performed by the Archimed Life Science GmbH laboratory (8,9). Tandem mass spectrometry-based detection of LysoGb3 was performed in positive ion mode (ES+) on a triple quadrupole mass spectrometer (Quattro Ultima, Waters, Milford, MA) with the NeoLynx software version 4.1. A multiple reaction monitoring mode was used for the measurement of LysoGb3. Patients with LysoGb3 levels of 3.5 ng/ml and above were considered to have high LysoGb3 levels in accordance with previous literature (10,11).

In patients with low αGLA enzyme activity, screening of GLA mutations was performed using DBS cards based on Sanger sequence analysis (Archimed Laboratory, Vienna, Austria). GLA mutations screened on nine patients with low enzyme activities were designed to identify the 600 mutations described for causing FD in the literature to date. SPSS 21.0 for Windows was used for the analysis of statistics. Descriptive statistics were presented as the number and percentage for categorical changes and as the average, standard deviation, and minimum, maximum, and median for numerical variations. When the numerical variations maintained the normal distribution conditions, the independent two groups were compared with the Student t-test, and when the numerical variations did not maintain the normal distribution conditions, the two groups were compared with the Kruskal-Wallis test. The subgroup analysis was performed with the Mann Whitey U test and interpreted with Bonferroni correction. The ratios in the groups were compared using Chi Square analysis. *p*<0.05 was considered as statistically significant.

## RESULTS

A total of 62 patients with biopsy-proven FSGS and 170 patients with ESRD undergoing HD were included in the study. Participants in the FSGS group were predominantly female (73.9%) with a mean age of 43.8±10.4 years (24-80 years), and the mean follow-up period was 9.3±3.48 years. Participants in the HD group were mostly men (55.3%) with a mean age of 61.7±13.9 years and mean HD length of 7.9±5 years. While the etiology of ESRD was unknown in 39% of the HD Group, the most commonly known causes were hypertension (28%) and diabetes (16%). Baseline values of median serum creatinine and median proteinuria of FSGS patients are shown in Table 1. Nine subjects (six in the FSGS group; three in the HD group) were detected as having low αGLA enzyme activity. The mean level of αGLA enzyme activity was found to be lower in FSGS patients than in the HD group (2.88±1.2 μmol/L/h *versus* 3.79±1.9 μmol/L/h, *p*<0.001). There was no statistically significant difference in the levels of plasma LysoGb3 between the groups (2.2±1.22 ng/ml *versus* 1.7±0.66 ng/ml, p: 0.4) (Table 1). In patients with low αGLA enzyme activities, one female patient’s GLA gene analysis showed a well-known single nucleotide polymorphism (SNP) at nucleotide C(937G>T) in exon p(D313Y) (Table 2). The laboratory values and basic characteristics of the patients with low αGLA enzyme activity are shown in Table 2.

The patient with this SNP was a 54-year-old female diagnosed with FSGS based on the results of a renal biopsy in 2014. She was referred to our hospital because of mild pretibial edema and 3+ proteinuria, per the results of a urine dipstick test. Her biochemical evaluation revealed that serum creatinine, proteinuria, and serum albumin were 0.95 mg/dl, 1.5 g/day, and 3.9 g/l, respectively. In the histopathological examination of a renal biopsy, 1 of a total of 35 glomeruli had global, and 3 had segmental sclerosis. Immunofluorescence staining for IgA, IgG, IgM, C3, C1q, fibrinogen, kappa, and lambda light chains yielded negative results. Electron microscopic examination could not be performed. She was treated with valsartan (160 mg/day) and methylprednisolone (60 mg/day). After 16 weeks with partial response to therapy, the doses of corticosteroids were tapered slowly over 8 months. The patient did not present any signs and symptoms of classical FD in the physical examination or her medical history. Left ventricular hypertrophy was not observed on transthoracic echocardiography with preserved ejection fraction (61%). Cardiac magnetic resonance imaging for findings suggestive of FD cardiomyopathy such as left ventricular structural changes, right ventricular hypertrophy, progressive myocardial fibrosis, or replacement fibrosis, showed no pathologic abnormalities. Cerebral MRI for cerebrovascular involvement was performed, and there were no signs of neurological involvement such as periventricular white-matter lesions or deep lacunar infarcts. Ophthalmological and dermatological examinations did not reveal any evidence in favor of FD. Extension of the genetic analysis to family members of the patient (her mother, her two sisters, her daughter, and her son) showed that none of them had any mutations or SNPs in the GLA gene. Four years after the diagnosis, clinical and laboratory parameters were stable.

## DISCUSSION

FD is an X-linked lysosomal storage disease that occurs because of deficient lysosomal αGLA enzyme activity. The genetic defect is maintained in all cell types, but the involvement of different organs and cell types differs broadly. Renal involvement results from Gb3 deposition in podocytes and the glomerular interstitial, mesangial, and endothelial cells. The epithelium of the loop of Henle, distal tubules, and the endothelial and smooth muscle cells of the renal arterioles get a share from this glycosphingolipid storage (12).

Sphingolipids are the main elements of the lipid structure of plasma membranes; they are important for podocyte functionality, and are indispensable for the glomerular filtration barrier (13). Globotriaosylceramide accumulation leads to progressive podocyte injury, vacuolar degeneration, and podocyte loss and is associated with segmental, and eventually, global glomerulosclerosis (14,15). Typical histopathologic findings in FD are glomerular basement membrane disorganization both at the podocyte and/or endothelial sites; interstitial fibrosis and tubular atrophy; focal, diffuse, global, and segmental glomerulosclerosis (6).

Proteinuria in FD is attributed to low αGAL enzyme activity and induces GL3 and LysoGL3 aggregation in podocytes and increase in autophagosome number, which causes podocyte depletion and contraction, and their dissociation from the glomerular basement membrane (16). Without clinically significant proteinuria, podocyte foot process effacement may be seen and is an indicator of podocyte stress or injury and also a characteristic marker of renal diseases with proteinuria (17). It may be accepted as an early sign of progressive FD nephropathy and FSGS (17,18).

Merscher et al. (13) defined a relationship between podocyte pathology and nephropathy. They identified FSGS and FD as diseases affecting the deposition of sphingolipids and sphingolipid metabolites. Sphingolipid accumulation may also be seen in some other glomerular diseases including lupus, HIV-associated nephropathy, and diabetic kidney disease. In this respect, FD and FSGS may share the same pathologic characteristics in many ways. Because of these similarities, a subgroup of FD patients with mild chronic histologic findings are misdiagnosed with primary FSGS and exposed to possibly harmful immunosuppressive therapies (19). Because of these pathologic similarities and treatment differences, we investigated αGLA enzyme activity in 62 biopsy-proven FSGS patients, and low enzyme activity was detected in 9% of the patients. However, none of the patients had typical symptoms of FD.

The reason for the patients’ low enzyme activity in FSGS could not be explained, and there is no information about whether these patients should be followed up or not in the literature. FD may coexist with various glomerular diseases, including immunoglobulin A nephropathy, granulomatosis with polyangiitis, crescentic glomerulonephritis, thin basement membranous nephropathy, lupus nephritis, and rheumatoid arthritis-associated renal diseases (14,20). There are no data about αGAL enzyme levels or activity in the normal population or in patients with glomerular diseases. Nakao et al. (8) found that nearly one percent of HD patients had low αGLA enzyme activity. A hereditary genetic alteration may not always be present, and low enzyme activity may be a part of the individual variation in its product or regulation. Therefore, our findings suggest that FD may be overestimated in general. Accordingly, enzyme activity may be decreased and Gb3 deposition may be seen in the aforementioned glomerular diseases, along with podocyte destruction and proteinuria.

More than 600 GLA gene mutations causing FD have been described in the literature (Human Gene Mutation Database, https://www.hgmd.cf.ac.uk). The pathogenicity of almost all alterations in exonic regions is supportive of the clinical observations. One example of such an SNP is the p. 'D313Y' substitution (G to T at cDNA nucleotide 937); while the plasma enzyme activity is remarkably decreased, high residual lysosomal enzyme activity and no pathologic excretion of urinary Gb3 were observed. The variant D313Y, with a prevalence of 0.5% in the general population, induces minimal or no clinical symptoms, with patients having a good prognosis and requiring no specific therapy (21-23).

Yasuda et al. (21) presented a 56-year-old male carrying the D313Y allele, with a deficient plasma αGLA enzyme level and without proteinuria or any other symptoms of FD. Electron microscopic evaluation showed that the interstitial, endothelial, arterial medial, and tubular cells, as well as the glomerular endothelial, mesangial cells, and podocytes were filled with the typical electron-dense LysoGL3 inclusions. Similarly, Oder D et al. (24) investigated six participants presenting with the D313Y haplotype, and they found no significant changes in any clinical and laboratory parameters between the baseline and fourth year. These results suggested that D313Y does not cause any organ involvement, but it might be a confounding factor with minimal symptoms, as a mild clinical variant of FD.

Most authors consider this mutation as a pseudodeficiency, but there are conflicting results in some related studies. For example, Gaspar et al. (25) reported a screening study including patients undergoing HD with hypertrophic cardiomyopathy for FD. They detected GLA alterations with deficient αGLA enzyme activities in seven patients, with two of them carrying a D313Y allele. Additionally, in another study including 493 young patients with stroke, 12 patients had missense GLA mutations, and D313Y was detected in 5 of these patients (26).

In light of these studies, we considered this SNP as a normal variation, because of the lack of diagnostic markers and typical symptoms of the disease. Moreover, according to the European Fabry Working Group consensus document, our patient did not meet the definite criteria for FD (11). We concluded that there was no need for enzyme replacement therapy.

The levels of many biomarkers such as thrombospondin-1 (TSP-1), transforming growth factor β1 (TGF-β1), vascular endothelial growth factor (VEGF), and fibroblast growth factor-2 (FGF2) are elevated in FD. Especially, TGF-β1 and VEGF have an impact on FD-associated glomerulosclerosis by inducing apoptosis and fibrosis in the kidneys of mice with FD. VEGF triggering results in TGF-β1-induced fibrosis in the proximal tubular cells. The expression of the extracellular matrix protein CD74 and TGF-β1 was enhanced by treating human podocytes with LysoGb3 (deacylated Gb3 form), showing that these are mediators of podocyte damage, and Gb3 is associated with inflammation and oxidative stress in FD (27). The levels of these cytokines and biomarkers have been reported to be elevated in focal segmental glomerulosclerosis and may affect steroid response in patients with FD (6).

This study has several limitations. Firstly, the low number of FSGS patients did not allow us to achieve a cut-off value for αGAL enzyme activity in this patient group. There is a need for a third control group comprising healthy individuals to be analyzed. Sanger sequence analysis can miss some deletions and mutations. The fact that the control group consisted of HD patients did not allow us to determine whether there was any relationship between the values of biochemical parameters of the patients and their αGAL enzyme activity.

In conclusion, we found that low enzyme activity may be detected in FSGS patients. More studies with larger cohorts are needed to reveal the link between these glomerular diseases and enzyme activity. We considered D313Y as an SNP and/or a normal variation. However, further investigation is also needed to address the conflicting studies about pD313Y mutations.

## AUTHOR CONTRIBUTIONS

Koc Y, Basturk T and Unsal A were responsible for the research idea and study design. Hasbal NB, Caglayan FB and Ucar ZA were responsible for the data acquisition. Hasbal NB, Sakaci T, Ahbap E and Sevinc M were responsible for the data analysis/interpretation. Hasbal NB and Caglayan FB were responsible for the statistical analysis. Hasbal NB and Caglayan FB were responsible for preparing the manuscript. Sevinc M, Koc Y, Basturk T, Sakaci T, Ahbap E and Unsal A were responsible for the supervision or mentorship.

## Figures and Tables

**Table 1:** Differences of age, sex, αGLA enzyme activity, and LysoGb3 between the groups.

	FSGS	HD	*p*
N	62	170	NA
Age, years	43.8±10.4	61.7±13.9	<0.001
Sex (Female, %)	73.9	44.7	<0.001
Baseline serum creatinine (mg/dl, median±SD)	1.01±0.35	NA	NA
Baseline Proteinuria (g/day, median±SD)	2.83±2.28	NA	NA
αGLA enzyme activity (µmol/l/h)	2.88±1.2	3.79±1.9	<0.001
LysoGb3 (ng/ml)	2.2±1.22	1.72±0.66	0.4

**Table 2:** Values of laboratory parameters and basic characteristics of the patients with low alpha-galactosidase A enzyme activity.

	Age	Gender	Group	αGLA Enzyme Activity (µmol/l/h)	LysoGb3 (ng/ml)	GLA Gene Alteration
Patient 1	50	Female	FSGS	0.9	3	No Mutation
Patient 2	54	Female	FSGS	1.1	3.1	D313Y
Patient 3	46	Male	FSGS	1.1	3.3	No Mutation
Patient 4	40	Male	FSGS	1.1	3.1	No Mutation
Patient 5	25	Male	FSGS	0.7	3.9	No Mutation
Patient 6	37	Female	FSGS	0.9	1.7	No Mutation
Patient 7	60	Male	ESRD	1.1	1.0	No Mutation
Patient 8	55	Male	ESRD	1.1	1.8	No Mutation
Patient 9	65	Female	ESRD	1.1	1.4	No Mutation
